# Genome-wide transcriptional response of *Escherichia coli* O157:H7 to light-emitting diodes with various wavelengths

**DOI:** 10.1038/s41598-023-28458-7

**Published:** 2023-02-03

**Authors:** Shehzad Abid Khan, Min-Jeong Kim, Hyun-Gyun Yuk

**Affiliations:** 1 4D Convergence Technology Institute, Korea National University of Transportation, 61 Daehark-Ro, Jeungpyeong-Gun, Chungbuk 27909 Republic of Korea; 2 Atta-ur-Rahman School of Applied Biosciences (ASAB), National University of Sciences and Technology (NUST), Islamabad, Pakistan; 3 Food Safety Risk Assessment Division, Food Safety Evaluation Department, National Institute of Food and Drug Safety Evaluation, Ministry of Food and Drug Safety, 187, Osongsaengmyeong2-Ro, Osong-Eup, Heungdeok-Gu, Cheongju-Si, Chungbuk 28159 Republic of Korea; 4 Department of Food Science and Technology, Korea National University of Transportation, 61 Daehark-Ro, Jeungpyeong-Gun, Chungbuk 27909 Republic of Korea

**Keywords:** Biotechnology, Computational biology and bioinformatics, Microbiology

## Abstract

We investigated the physiological and transcriptomic response of *Escherichia coli* at the early stationary phase to light-emitting diodes with different wavelengths. The growth and metabolic changes of *E. coli* O157:H7 were examined under the influence of 465, 520, and 625 nm illuminated light. Under 465 nm illumination, the growth of *E. coli* O157:H7 was significantly retarded compared to 520 nm and 625 nm illumination and non-illuminated control. Metabolic changes were examined under these illumination and non-illuminated conditions based on transcriptomic reads. Transcriptomic response under 520 nm and 625 nm remained almost similar to control except few up-and down-regulated genes. Carbohydrates metabolic transcriptomic reads were greatly down-regulated under 465 nm illumination compared to 520 nm and 625 nm illumination and non-illuminated control showing depletion of glucose as a sole energy source during the exponential phase. Fatty acid degradation such as *fad* regulon-related genes was up-regulated in cells under 465 nm illumination revealing the shifting of cells to use fatty acid as a new carbon energy source during the early stationary phase. Exposure of *E. coli* O157:H7 cells to 465 nm illuminated light down-regulated virulence factor genes such as *hlyA*, *hlyB*, *hlyC*, *stx1A*, *stx2B*, *paa*, and *bdm*. Under the stress of 465 nm illumination, expression of stress and flagellar motility-related genes were up-regulated causing consumption of energy and reduction in cell growth. Also, oxidative phosphorylated transcriptomic reads were up-regulated under 465 nm illumination probably due to the production of ROS that might involve in the reduction of cell growth during the early stationary phase. These results indicate that pathogenic *E. coli* O157:H7 respond differentially to a different wavelength of the light-emitting diodes used in this study.

## Introduction

Indoor plant production with artificial light emitting diodes is of great interest these days with consideration of the production of organic vegetables in a clean precise control environment and combat of land resources and environmental factors^1^. Different kinds of vegetables such as tomatoes, potatoes, chilies, cabbages, and lettuces have been grown successfully in indoor factories^1^. Light, temperature, humidity, air, and nutrition are the most essential factors for plant growth. Indoor plant factories under control environments have a higher potential for production and advantages compared to traditional horticulture. As climate change has already been reported to be involved in great food production loss^2,3^. Additionally, climate change with natural disasters has a negative impact on major agricultural crop production such as maize crop production in Northeast China reduced by half from 1997 to 2017^4^. It is estimated that these extreme weather changes may lead to severe food shortages and hunger for 170 million people by 2080^5,6^.

In order to meet food shortage, indoor plant production is considered the best alternative approach that needs artificial lights for the photosynthesis of plants. Among artificial lights, light emitting diodes (LED) consider the best option having several advantages such as lack of low-pressure mercury lamps (LPM), small size, long life, non-thermal and can also be used efficiently to increase nutritional values, and control the microbial population in plants and vegetables^7,8^. The effect of LEDs with different wavelengths has been studied previously to investigate their effects on vegetables and fruits. Such as 660 nm LEDs were effective for the predominant accumulation of carotenoid (*β*-cry) in Satsuma mandarins^9^. Furthermore, blue (465 nm) and red (625 nm) LEDs on pea seedlings increased the concentration of chlorophyll and *β*-carotene contents^10^.

Fresh-cut produce production has increased to 64.8% in Korea during the last decade^11^. Similarly, the meal kits industry is also flourishing worldwide, a growth of 300% was noticed in the United State (US) in 2017 valued at 4.65 billion US dollars^11^. Both fresh-cut produce from indoor plant factories and meal kits contain various vegetables that usually consume without processing. *Escherichia coli* O157:H7 is known as the most common pathogen in fresh produce and cause diseases in human such as hemorrhagic colitis, bloody diarrhea, and hemolytic uremic syndrome^12^. From 2008 to 2020 a total of 515, 165, and 235 cases of foodborne outbreaks from fresh produce were reported due to enterohemorrhagic *E. coli* (EHEC) in the US, the United Kingdom (UK), and Canada, respectively^13–15^. Until now, no outbreak has been reported at the indoor plant factories, however, with the advancement and necessity of indoor plant factories, chances of contamination of vegetables due to *E. coli* O157:H7 have also increased.

To reduce foodborne diseases, especially the inactivation of *E. coli* O157:H7 in fresh produce, a sanitizing step is essential. Washing baby leaves, vegetables or soft fruits could change their shape and appearance because of their fragile structure and would lead to the loss of their commercial value. Fresh produce might have high microbial growth without washing steps after the post-harvesting period^16,17^. One method to kill microbes is ultraviolet (UV) light used with LPM that interferes with DNA replication and leads to microbial cell death^18^. However, UV irradiation is not encouraged in the food industry because of its serious physical and chemical hazards^19^. LED emerge as a potential alternative to other treatments and use in the surface treatment of fresh produce. Studies have shown that UV-LEDs have the ability to control microbial growth in different products of fruits and vegetables^20^. Based on composition and semiconductor material, LED can be designed to emit the desired wavelength^19^. The antibacterial effect of LEDs with different wavelengths was investigated against foodborne pathogens and found blue (461 nm) and green (521 nm) LEDs were effective in controlling these pathogens^21,22^.

The inhibition effects of LEDs of different wavelengths against *E. coli* O157:H7 have been investigated previously on fresh produce^23^. Studies showed that intracellular molecules in bacteria absorb light wavelengths that affect their growth^22^. However, the genome expression changes based on transcriptomic sequences of *E. coli* O157:H7 under treatment of different wavelengths have not been reported previously. Transcriptomic sequences provide useful insight to examine the changes in genomic and metabolic features of a single microbial species while comparing various environments including different wavelength stress. Therefore, in this study, we examined the growth of *E. coli* O157:H7 under the stress of different wavelength light, extracted their total RNAs, and sequenced them to understand their response to long-term exposure to blue, green, and red LED illumination at the molecular level.

## Results

### Effect of LED illumination on microbial growth

Three different LEDs (blue, green, and red) were found to have intensity peaks at 465, 520, and 625 nm wavelengths, respectively (Table 1). Since LED illumination for a long time could increase the temperature of the growth medium, the temperature of TSB was monitored for 4 h during LED illumination to select the optimum temperature condition for cell growth under LED illumination. Regardless of wavelength, LED illumination resulted in about a 0.5 °C increase in TSB temperature, compared with the set temperature of the incubator (data not shown). Thus, the temperature for cell growth under LED illumination was adjusted to 24.5 °C to eliminate the temperature effect on cell growth.

**Table 1:** Specification of high intensity light-emitting diodes (LED).

Color	Range of wavelength (nm)^§^	Range of luman (lm)	Voltage (v)	Electric current (mA)
Blue	460–470 (465)	200–300	9–11	1050
Green	515–525 (520)	600–800	9–11	1050
Red	620–630 (625)	500–600	8–10	1200

^§^The wavelength in the parentheses is the highest peak wavelength of each LED.

**Figure 1:**
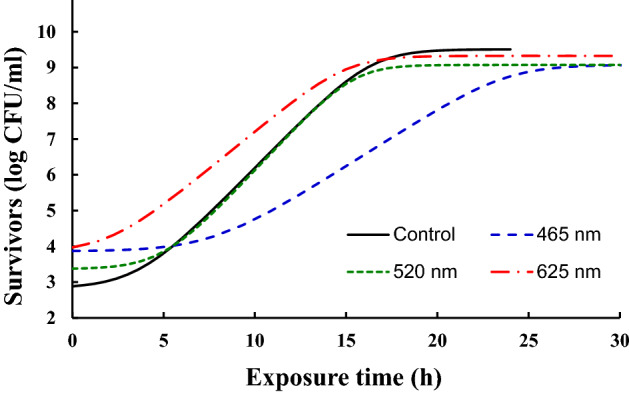
Growth survival of *E. coli* O157:H7 under the influence of different wavelengths light. Cells growth under dark condition was served as control.

The average growth curves for *E. coli* O157:H7 cells grown under dark condition or each LED illumination as fitted to the Baranyi model (Fig. 1). The coefficient of determination (R^2^ values) for the fitted growth curves were greater than 0.99 (data not shown). The growth pattern of *E. coli* O157:H7 was altered by LED illumination with different wavelengths. The cell growth under dark condition (control) was similar to that of 520 nm, while the growth patterns of cells during 465 and 625 nm LED illumination were different from that of control cells in TSB at 25 °C. The growth parameters of non- and LED-illuminated *E. coli* O157:H7 were calculated based on the fitted growth curves (Table 2). There were no significant (*P* < 0.05) differences in LPD values between control and 520 nm, and control and 625 nm, respectively, whereas cells grown under 465 nm illumination had longer lag phases than the others. Similarly, lower GR and higher DT were observed in cells grown under 465 nm illumination than those of control cells. In addition, cells under 465 nm illumination reached significantly (*P* < 0.05) lower MPD compared to that of the control. Unlike 465 nm, no significant differences in DT and MPD values between the control and cells are grown under 625 nm illumination. These results indicate that cell growth was highly influenced by 465 nm LED illumination, while LEDs of other wavelengths did not. On the basis of the growth curves, the early stationary phase was determined as 17 h for non-illuminated control and cells under 625 nm illumination, whereas it was reached at 18 h and 29 h for cells under 520 nm and 465 nm illumination, respectively. The collected cells at the early stationary phase were subjected to RNA-seq analysis.

**Table 2:** Growth parameters of non- and LED-illuminated *E. coli* O157:H7 in TSB at 25 °C.

Illumination	LPD (h)	GR (h)	DT (h)	MPD (log CFU/ml)
Control	3.9 ± 1.0^bc^	0.6 ± 0.1^a^	1.3 ± 0.2^b^	9.5 ± 0.2^a^
465 nm	7.9 ± 1.0^a^	0.3 ± 0.1^c^	2.2 ± 0.3^a^	9.0 ± 0.1^c^
520 nm	5.5 ± 0.4^b^	0.5 ± 0.1^ab^	1.6 ± 0.4^b^	9.1 ± 0.2^bc^
625 nm	2.6 ± 0.6^c^	0.4 ± 0.0^bc^	1.7 ± 0.1^b^	9.3 ± 0.1^ab^

Different letters for the same column indicate significant difference (*P* < 0.05) difference.

*LPD* lag phase duration, *GR* specific growth rate, *DT* doubling time, *MPD* maximum population density.

### Changes in transcriptome

**Figure 2:**
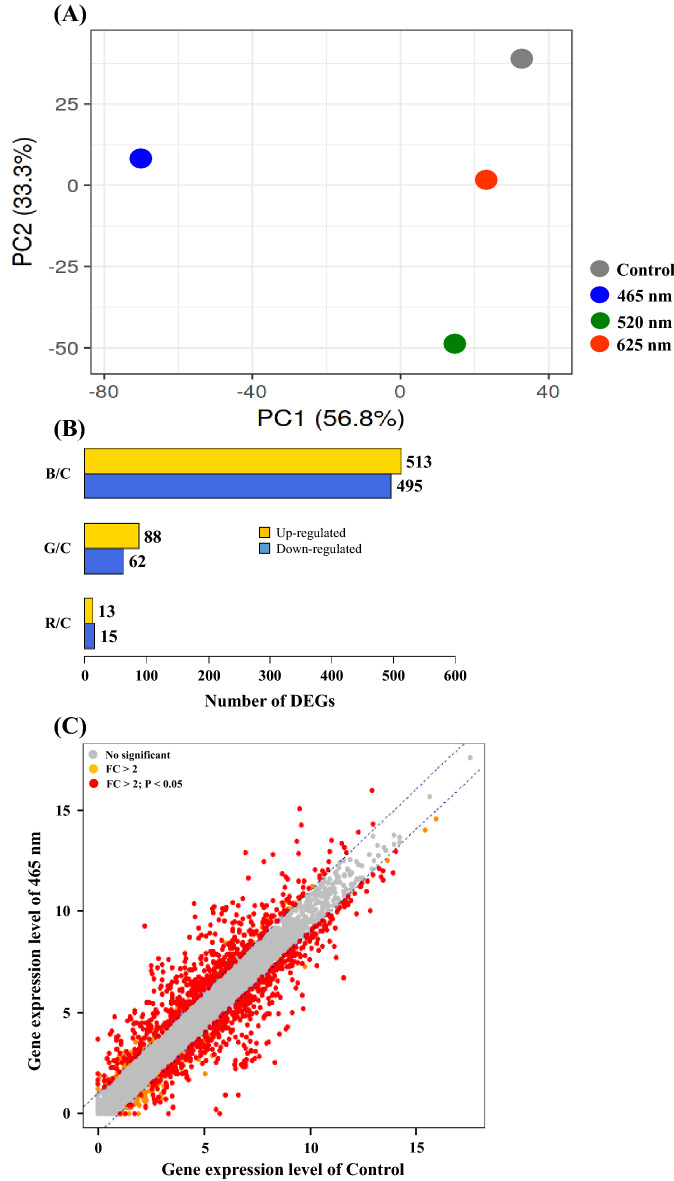
Principal component analysis (PCA) of genes expression of *E. coli* O157:H7 treated under different wavelength of lights. Cells growth under dark condition was served as control. Unit variance scaling is applied to rows; SVD with imputation is used to calculate principal components. X and Y axis show principal component 1 and principal component 2 that explain 56.8% and 33.3% of the total variance, respectively. N = 4 data points (**A**), Distribution of differentially expressed genes (DEGs). The X-axis is the comparison of different groups (B, 465 nm; G, 520 nm; R, 625 nm; C, control) and the Y-axis is the number of DEGs (**B**) and Scatter plot to show the distribution of DEGs. The gray color represents non-DEGs and genes scattered along X–Y axis indicate the genes which were overabundant under blue 465 nm illuminated light and Control (**C**).

The PCA was performed to examine the similarities and differences in transcriptomic reads among samples under 465, 520, and 625 nm illumination and control (Fig. 2A). The maximum gene variations in these 4 groups were 56.8% (PC1) and 33.3% (PC2) with an acceptable separation and cluster formation, illustrating that the genes of *E. coli* O157:H7 reacted differently to 465 nm, 520 nm, 625 nm illumination, and control. Moreover, transcriptomic reads of cells treated under 520 nm and 625 nm illumination were closer to those of control, while they were totally different from transcriptomic reads of cells treated under 465 nm illumination, exhibiting that 465 nm illumination might induce great transcriptome changes in *E. coli* O157:H7 cells compared with non-illuminated control and 520 nm and 625 nm illumination. The DEGs under illumination conditions were also compared with control with an adjusted fold change (FC) > 2 and *P* < 0.05 (Fig. 2B). Under 465 nm LED illumination, a higher proportion of genes were over-expressed (513 genes) and under-expressed (495 genes) compared to 520 nm (88 up-regulated; 62 down-regulated genes) and 625 nm (13 up-regulated; 15 down-regulated genes) LED illumination in comparison with control. Additional gene expression levels under 465 nm illumination and control were compared and visualized as scatter plots with an adjusted FC > 2 and *P* < 0.05 (Fig. 2C). Scatter plot presents the significance and differences in transcriptomic reads. Additionally, the transcriptional response of *E. coli* O157:H7 under different LED illumination was assessed (Table 3). The results showed significant up-or down-regulation of genes in *E. coli* O157:H7 cells. The highest number of genes in which the expression was significantly affected was caused after exposure of *E. coli* O157:H7 to 465 nm LED illumination, however, for the control, 520 nm and 625 nm illumination, the number of genes significantly up-or down-regulated was quite similar. The 465 nm LED illumination also significantly downregulated genes related to virulence factors (*hlyA*, *hlyB*, *hlyC*, *hlyE*, *stx1A*, *stx2A*, *paa*) and flagellar proteins (*csgF*, *csgC*, *fimC*, *fimD*) in comparison with the control, 520 nm and 625 nm LED illumination.

**Table 3:** Genes up- and/or down-regulation in *E. coli* O157:H7 under 465 nm, 520 nm and 625 nm LED illuminated and non-illuminated control cells.

Gene symbol	Gene description	Control (log2)	465 nm (log2)	520 nm (log2)	625 nm (log2)
Virulence factors					
* hlyA*	Hemolysin protein A	5.78 ± 0.65	4.76 ± 2.65*	5.78 ± 1.03	5.74 ± 2.74
* hlyB*	Hemolysin protein B	3.43 ± 0.44	2.67 ± 0.02*	3.38 ± 0.83	3.14 ± 3.5
* hlyC*	Hemolysin protein C	5.24 ± 2.69	4.26 ± 2.43*	5.16 ± 1.01	5.04 ± 3.41
* hlyD*	Hemolysin protein D	3.55 ± 0.04	3.13 ± 1.27	3.34 ± 1.10	3.25 ± 0.96
* hlyE*	Hemolysin protein E	7.08 ± 5.14	6.72 ± 3.74	7.62 ± 3.85*	7.55 ± 3.42*
* stx1A*	Shiga-like toxin 1 subunit A	8.73 ± 4.86	8.07 ± 4.76*	8.65 ± 6.04	8.57 ± 4.02
* stx1B*	Shiga-like toxin 1 subunit B	9.42 ± 4.60	9.49 ± 5.93	9.63 ± 7.20	9.32 ± 4.05
* stx2A*	Shiga-like toxin II subunit A	3.39 ± 1.68	2.75 ± 0.80	3.46 ± 1.61	3.22 ± 1.31
* stx2B*	Shiga-like toxin II subunit B	6.79 ± 3.76	6.20 ± 2.89*	7.07 ± 4.93	6.95 ± 4.18
* paa*	Bacterial adherence	5.75 ± 3.30	4.39 ± 1.50*	5.98 ± 3.71	5.92 ± 2.74
* bdm*	Biofilm-dependent modulation protein	6.77 ± 0.95	6.23 ± 2.21*	6.39 ± 4.05	6.56 ± 4.99
Flagellar protein					
* csgF*	Curli assembly protein D	4.29 ± 4.17	2.04 ± 0.40	3.57 ± 3.38	3.61 ± 3.40
* csgC*	Curli assembly protein C	2.75 ± 3.26	0.26 ± 0.47	3.00 ± 3.66	3.37 ± 3.97
* fimC*	Chaperone protein C	2.76 ± 2.01	1.61 ± 0.97	0.70 ± 0.48	1.20 ± 0.19
* fimD*	Outer membrane usher	1.87 ± 1.36	0.76 ± 1.87*	2.21 ± 1.65	2.11 ± 2.67
Fatty acid degradation					
* fadL*	long-chain fatty acid transporter	5.97 ± 4.57	6.12 ± 1.59	4.75 ± 2.21	5.28 ± 3.47
* fadJ*	3-hydroxyacyl-CoA dehydrogenase	5.21 ± 1.73	6.53 ± 3.35*	4.85 ± 2.63	5.22 ± 1.36
* fadI*	3-hydroxyacyl-CoA dehydrogenase	4.82 ± 1.61	5.64 ± 2.90*	4.11 ± 2.06*	4.24 ± 2.52
* fadH*	2,4-dienoyl-CoA reductase (NADPH)	-0.19 ± 2.14	3.05 ± 1.07	0.41 ± 0.77	0.01 ± 2.37
* fadE*	long-chain-acyl-CoA dehydrogenase	3.68 ± 0.15	4.97 ± 2.93	3.41 ± 1.37	3.78 ± 1.36
* fadD*	acyl-CoA synthetase	4.66 ± 2.11	5.73 ± 2.84*	4.10 ± 2.13	4.39 ± 1.23
* fadB*	oxidation complex	1.44 ± 1.94	2.92 ± 0.98	1.43 ± 0.39	1.77 ± 1.04
* fadA*	3-ketoacyl-CoA thiolase FadA	3.50 ± 1.37	3.77 ± 0.70	2.86 ± 1.46	3.35 ± 1.70

*Indicate significant difference (*P* < 0.05) between 465 nm; 520 nm and 625 nm illuminated light in comparison with control.

### Transcriptomic analysis of *E. coli* O157:H7 under LED-illumination based on pathways from the KEGG database

**Figure 3:**
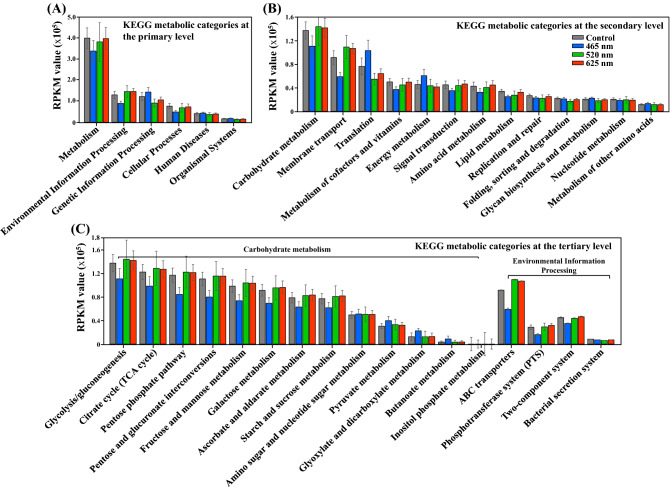
*Escherichia coli* O157:H7 transcriptional expression (RPKM, read numbers per kilobase of each coding sequence, per million mapped reads) of representative KEGG functional categories at the primary (**A**) secondary (**B**) and tertiary (**C**) levels treated under different wavelength of lights. Cells growth under dark condition was served as control.

To examine the metabolic features of *E. coli* O157:H7, the strain was cultivated under LED-illumination and non-illuminated conditions, and the transcriptome was analyzed. The functional genes relative activities were calculated through the relative abundance of *E. coli* O157:H7 mRNA reads from the total number of mRNA reads of *E. coli* O157:H7. The mRNA reads of *E. coli* O157:H7 were functionally assigned to each KEGG metabolic category (Fig. 3). The KEGG distributions of the *E. coli* O157:H7 mRNA reads under 465 nm LED illumination were different from non-illuminated control and 520 nm and 625 nm LED illumination. The mRNA transcripts at the primary level were predominantly assigned to the metabolic category under all of the four conditions (Fig. 3A). At the secondary level, mRNA transcripts in the case of non-illuminated control and 520 nm and 625 nm LED illumination were predominantly assigned to the carbohydrate metabolism category, however, in case of 465 nm LED illumination, the mRNA transcripts were equally predominantly assigned to carbohydrate metabolism and translation categories as well (Fig. 3B). In case of 465 nm LED illumination, mRNA reads for carbohydrate metabolism were decreased, resulting in the delayed growth of *E. coli* O157:H7. In the case of non-illuminated control and 520 nm and 625 nm illumination, the second-most abundant mRNA reads were assigned to membrane transport category and significantly higher than the mRNA reads under 465 nm illuminated *E. coli* O157:H7 (Fig. 3B). In the case of non-illuminated control and 520 nm and 625 nm LED illumination, the second-most abundant mRNA reads were assigned to the membrane transport category and significantly higher than the mRNA reads under 465 nm LED illuminated *E. coli* O157:H7 (Fig. 3B). In addition, mRNA reads of *E. coli* O157:H7 at the tertiary level showed the subcategories of carbohydrate metabolism and environmental information processing categories (Fig. 3C). Environmental information processing genes mainly involved in ABC transporters and PTS system pathway, which are involved in carbohydrate metabolism and respond to environmental system conditions were down-regulated under 465 nm LED illumination. In almost all of the categories at the tertiary level, mRNA reads in the case of 465 nm LED illumination were significantly decreased as compared to non-illuminated control and 520 nm and 625 nm LED illumination.

**Figure 4:**
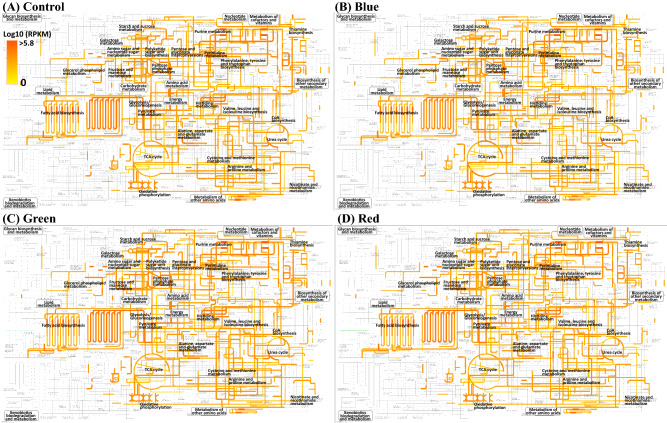
Transcriptional expression of the metabolic pathways of *E. coli* O157:H7 treated under different wavelength of lights. Cells growth under dark condition was served as control. The KEGG metabolic pathways were generated using the genome of strain *E. coli* O157:H7; their transcriptional expression levels are quantitatively depicted using line thickness and color changes, according to their read numbers per kilobase of each coding sequence, per million mapped reads (RPKM) values.

**Figure 5:**
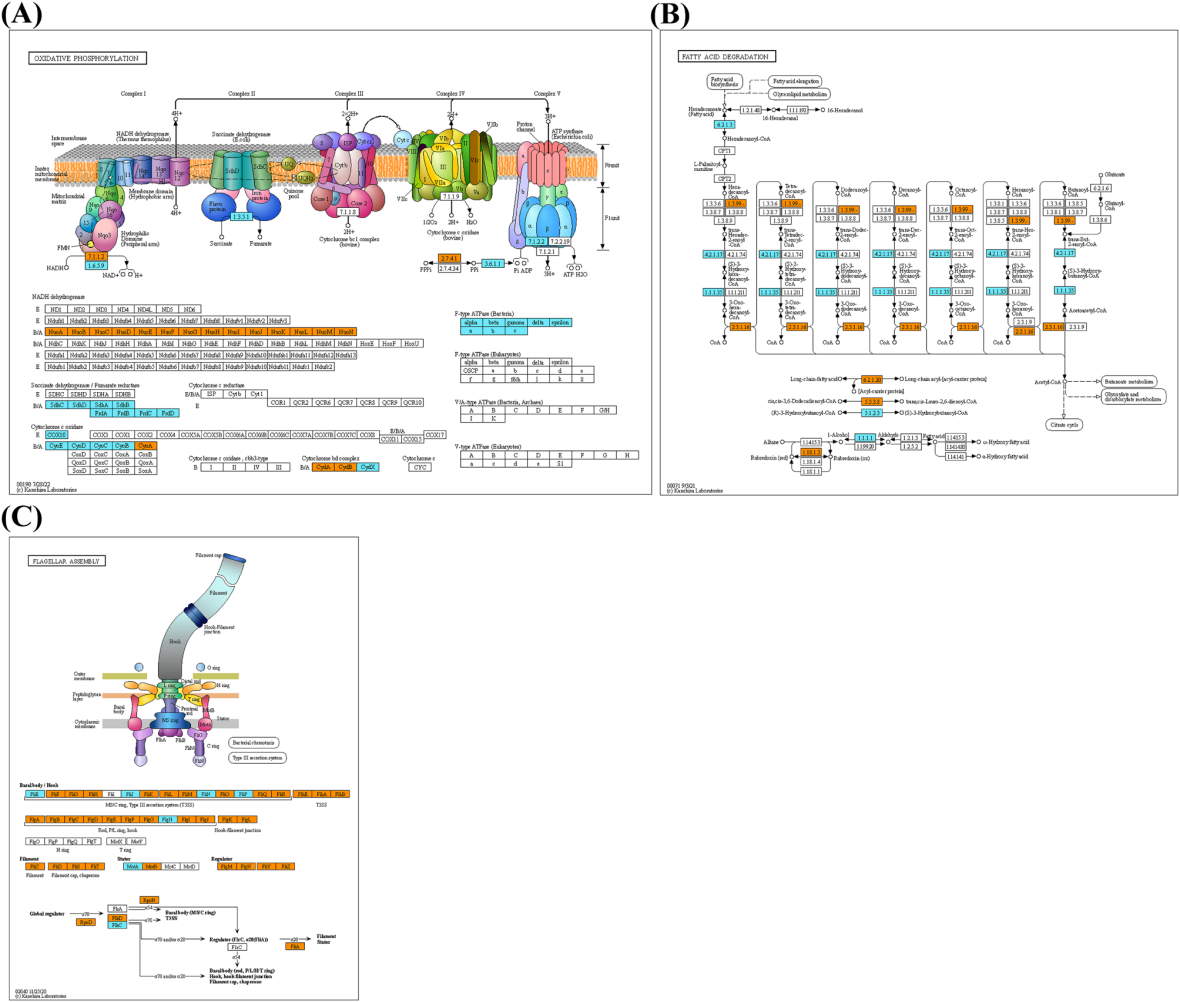
Down-regulated (blue) and up-regulated genes (Orange) of *E. coli* O157:H7 under the influence of blue (465 nm) illuminated light in comparison with the control in the pathway of oxidative phosphorylation (**A**), fatty acid degradation (**B**) and flagellar assembly (**C**). Pathway analysis of the selected genes was performed using the KEGG database.

The metabolic features of *E. coli* O157:H7 were further examined by mapping mRNA reads of cells under different LED-illuminated and non-illuminated lights to the KEGG pathways (Fig. 4). The KEGG metabolic transcriptomic analysis showed that some metabolic pathways of *E. coli* O157:H7 under 465 nm LED illumination were also up-regulated such as those involved in translation and energy metabolism. However, many other genes, such as metabolic pathways involved in carbohydrate metabolism and membrane transport, were down-regulated in comparison with non-illuminated control and 520 nm and 625 nm LED illumination. A variety of transcriptomic reads related to oxidative phosphorylation (Fig. 5A), fatty acid degradation (Fig. 5B), and flagellar assembly (Fig. 5C) were up-regulated in cells under 465 nm LED illumination as compared to transcriptomic reads of *E. coli* O157:H7 under non-illuminated control. Such as in oxidative phosphorylation genes *nuoA* to *nuoN* encoding an NADH: ubiquinone oxidoreductase was up-regulated, however, cytochrome o oxidase complex encoding genes *cyoBCDE* were down-regulated upon exposure of cells under 465 nm LED illumination. Several dehydrogenase genes were also down-regulated upon exposure to 465 nm LED illumination, for example, succinate dehydrogenase encoding genes *sdhABCD* and fumarate reductase encoding genes *frdABCD*. These genes involve in reducing ubiquinone to ubiquinol, which donates an electron to terminal oxidases, cytochrome o, or the cytochrome d complexes that oxidase ubiquinol and reduce molecular oxygen to water. The down-regulation of electron transport chain (ETC) components illustrated that cells under 465 nm illumination were not healthy enough for aerobic respiration compared to non-illuminated healthy control cells (Fig. 5A). It seems that cells consume fatty acids as sole carbon and energy sources with various chains. *E. coli* cells uptake fatty acids and degrade via the *β*-oxidation pathway or use fatty acids for the biosynthesis of membrane phospholipids. Enzyme *fad* regulon involve in catalysis of these degradation pathway that activate and transport of long chain fatty acid and degrade into acetyl CoAs (Table 3, Fig. 5B). In the present study, genes *fadL, fadJ, fadI, fadH, fade* and *fadD* involved in acetyl CoAs production were significantly up-regulated to 6.12, 6.53, 5.64, 3.05, 4.97 and 5.73 log2, respectively, under 465 nm LED illumination compared to 520 nm (4.75, 4.85, 4.11, 0.41, 3.41 and 4.10 log2, respectively) and 625 nm (5.28, 5.22, 4.24, 0.01, 3.78 and 4.39 log2, respectively) illuminated and non-illuminated control cells (5.97, 5.21, 4.82, − 0.19, 3.68 and 4.66 log2, respectively) (Table 3, Fig. 5B). The motility encoding genes were up-regulated in cells under 465 nm LED illumination compared to control cells (Fig. 5C). As the flagellum master regulator gene *flhD*, genes *fli*, and *flg* are involved in the regulation, biosynthesis, and assembly of flagellum, and motor complex protein *motB* was upregulated in *E. coli* O157:H7 upon exposure to 465 nm illumination.

## Discussion

Exposure of *E. coli* O157:H7 cells to different wavelengths of LEDs induced transcriptional changes as described in the present study. Previously, the effect of LEDs was investigated against pathogenic bacteria, and found blue (461 nm) and green (521 nm) LEDs were effective in controlling these pathogens^21,22^. In the present study, three different LED-illuminated lights were used to examine their effects on bacterial growth, and found *E. coli* O157:H7 cells respond differentially to 465 nm illuminated light as described by Ghate et al.^21^. These findings could be elaborated by the photodynamic treatment mechanism involved in bacterial inactivation. The photodynamic treatment induces the excitation of photosensitizer molecules that produce reactive oxygen species (ROS) when absorbing wavelengths between 400 and 500 nm^21,24^. The ROS then oxidize the cell membrane constituents and cause cytotoxic effects^21,25^.

Based on transcriptional differences in *E. coli* O157:H7 under the influence of different LEDs illuminated light affected the growth spectrum (Fig. 1). Therefore, transcriptomic reads of *E. coli* O157:H7 grown under these conditions were compared. A significant number of DEGs were observed for *E. coli* O157:H7 grown under 465 nm illumination compared to 520 nm and 625 nm illumination and non-illuminated control (Fig. 2). From *E. coli* O157:H7, growth spectrum under different LED-illuminated light, we assumed LED lights affected on DNA, proteins or lipids. Based on transcriptomic reads of *E. coli* O157:H7 under illuminated and non-illuminated conditions, KEGG metabolic categories were analyzed (Fig. 3). However, no significant differences in RPKM values were observed for amino acid and lipid metabolism (Fig. 3B). Therefore, protein and lipid oxidation are not sufficient to elaborate different growth levels of *E. coli* O157:H7 grown under different stress conditions. Based on KEGG analysis, biological, metabolic, and cellular processes have the largest number of DEGs.

Metabolic processes of *E. coli* O157:H7 at the initial stationary phase could be identified based on KEGG pathways, which show an interacting molecules network (Fig. 4). In bacterial cells, ATP-binding cassette (ABC) transporters proteins contain two transmembrane domains (TMDs) and two nucleotide-binding domains (NBDs)^26^. The NBDs involve in binding and hydrolyzing ATP and structural changes in TMDs for conduit opening to transport substrates^27^. In *E. coli* cells, 5% of the total genome is represented by the largest protein family of ABC transporters containing 80 diverse systems^28^. In our study, transcriptomic reads encoding ABC transporters systems under influence of 465 nm illuminated light were down-regulated compared to others such as genes involved in oligopeptide transport system (*oppB*, *oppC*, *oppF*), lipoprotein-releasing and transport system (*lolA*, *lolB*, *lolC*, *lolD*), histidine transport system (*hisP*) and lysine/arginine/ornithine transport system (*argO*). These extracellular binding proteins facilitate the transportation of substrates into the cell^29^. The down-regulation of these ABC transporter genes might change the importing function of the membrane to uptake nutrients for cell growth under exposure to 465 nm illuminated light and could be the reason for suppressed growth of *E. coli* O157:H7 cells compared to cells grown under 520 nm and 625 nm illumination and non-illuminated control.

Additionally, a series of enzymes are involved in the reduction and oxidation of glucose to acetyl-CoA and finally into carbon dioxide with the production of energy. The pathway converts NAD^+^ and FAD into NADH and FADH_2_, respectively, with the production of one GTP. Then in the oxidative phosphorylation pathway, this NADH and FADH_2_ produce ATPs^30^. In our study, based on transcriptomic reads, genes involved in the glucose metabolic pathway mainly glycolysis, TCA cycle, pentose phosphate, pentose and glucuronate interconversions, fructose and mannose, galactose, ascorbate and aldarate, and starch and sucrose metabolism genes were significantly down-regulated in *E. coli* O157:H7 grown under 465 nm illumination compared to 520 nm and 625 nm illuminated and non-illuminated control, that showed the less production of NADH and FADH_2_ and energy depletion during carbohydrate metabolism in exponential phase (Fig. 3).

However, fatty acid metabolism produced a high amount of energy than other carbon sources because of its availability as a high reduction and less oxygenation state and plays a vital role in bacterial adaptation during the stationary phase^31^. Genes related to fatty acid metabolism and degradation were upregulated in *E. coli* O157:H7 grown under 465 nm illumination compared to 520 nm and 625 nm illumination and non-illuminated control. These genes are involved in the *β*-oxidation cleavage of long-chain fatty acids into acetyl CoAs. Firstly, an outer membrane-associated protein *fadL* and inner membrane acyl-CoA synthase *fadD* activate acyl-mechanism involve in the transportation of long-chain fatty acids across the bacterial cell membrane, and then *fadE* converts acyl-CoA to enoyl-CoA^32^. A tetrameric complex made up of two copies of *fadB* and *fadA* completes the final stages of fatty acid degradation, which include hydration, oxidation, and thiolytic cleavage. The *β*-oxidation pathway functions in a cyclic manner, shortening the input acyl-CoA by two carbon atoms to produce acetyl-CoA after each cycle. The results showed that *E. coli* O157:H7 under influence of 465 nm illuminated light, might use fatty acids as an alternative energy source to survive.

Also, under the stress of 465 nm illuminated light, *E. coli* O157:H7 might use a flagellar motility system for survivability and adaptation to favorable conditions. Bacteria use flagellar motility in response to environmental stimuli such as pH, temperature, various chemicals, redox potential, and osmolarity that enable bacterial cells to adopt favorable environments for growth and survival^33^. For motility, *E. coli* cells consume enough cellular protein and energy for the biogenesis of the flagellar motility system^34,35^. Expression of motility-related genes effect on reduction of bacterial growth as flagellar motility consumes enough amount of nutrient contents and cellular protein required for bacterial rotation^36,37^. In our study, we observed the transcriptomic reads of *E. coli* O157:H7 under 465 nm illumination related to flagellar motility greatly upregulated in comparison with non-illuminated control (Fig. 5C). This upregulation of motility related genes might be responsible for bacterial growth reduction, down-regulation of carbohydrates metabolic genes and upregulation of fatty acid degradation genes.

Besides this, transcriptomic data also showed that LED lights also affect the quorum sensing (QS) capability of *E. coli* O157:H7. Bacterial cells communicate through QS and monitor cell density population based on signaling molecules concentration in the surrounding environment and express genes accordingly^38,39^. The autoinducers-2 (AI-2) signal molecule is an inter-species signaling system encoded by the *luxS* gene that inter-converts AI-2 molecules to 4,5-dihydroxy-2,3-pentanedione (DPD)^38,40^. Our study showed that transcriptomic reads of the *luxS* greatly down-regulated under 465 nm illuminated light compared to others. Pathogenic strains use QS to regulate virulence factors and the down-regulation of the *luxS* gene demonstrated that communication between cells under 465 nm illuminated light might decrease and virulence potential as well.

## Conclusion

In conclusion, 465 nm illuminated blue light greatly reduced the growth of *E. coli* O157:H7 in comparison with 520 nm and 625 nm illuminated and non-illuminated control. The results of DEGs showed that a few genes of *E. coli* O157:H7 respond differentially under 520 nm and 625 nm illuminated light compared to non-illuminated control groups. Gene expression of *E. coli* O157:H7 greatly changed under 465 nm illuminated light, specifically, genes related to glucose metabolism were significantly down-regulated, causing up-regulation of fatty acid degradation and oxidative phosphorylation genes during the early stationary phase. Additionally, the motility-related genes were also up-regulated to adapt to favorable niches and survive under the stress of 465 nm LED illumination. Overall these results could help to understand the response of *E. coli* O157:H7 under the wavelength of different LEDs. Comparative analysis of different wavelengths with the non-illuminated control group based on transcriptomic reads revealed that metabolic activities of *E. coli* O157:H7 were significantly affected under illuminated conditions.

## Materials and methods

### Bacterial strain and culture conditions

*E. coli* O157:H7 (C7927; apple cider isolate) obtained from Dr. Kun-Ho Seo at Konkuk University, Republic of Korea was used in this study as a model strain since it was isolated from fresh produce. A frozen culture of *E. coli* O157:H7 was activated by incubation for 24-h at 37 °C in 10 ml sterile tryptone soya broth (TSB; Oxoid, Basingstoke, Hampshire, UK). The culture was centrifuged at 3500×*g* for 10 min at 4 °C and washed twice with phosphate-buffered saline (PBS; Biosesang, Seongnam-si, Korea). The cells in the resultant pellet were resuspended in 1 ml of PBS and serially diluted to approximately 10^4^ CFU/ml using PBS. The last serial dilution of 10^4^ CFU/ml was made in 10 ml sterile TSB for LED illumination.

### Light-emitting diode (LED) illumination

**Figure 6:**
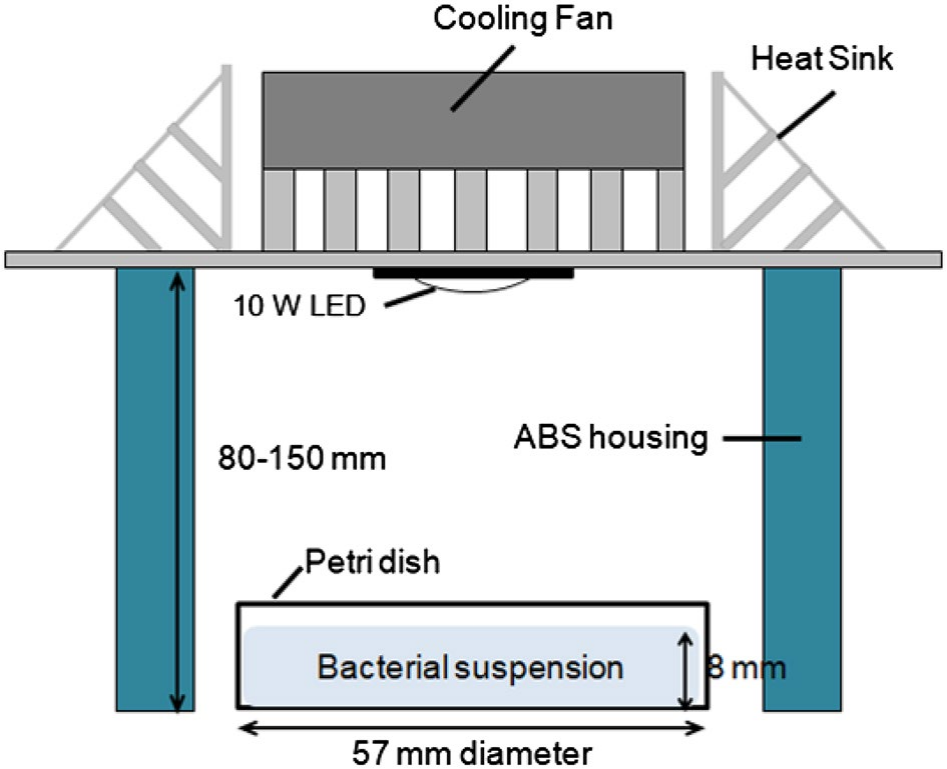
Experimental setup to check the growth of *E. coli* O157:H7 under the influence of different wavelengths light.

High-intensity LEDs (10-W) with wavelengths of 465, 520, and 625 nm were purchased from Shenzhen Getian Opto-Electronics Co., Ltd. (Shenzhen, Guangdong, China). The specification of each LED was described in Table 1. LEDs (8 by 8 mm) were attached to a heat sink and a fan to reduce heat transfer to the cell suspension. Each LED system was surrounded with an acrylonitrile butadiene styrene (ABS) housing to prevent the penetration of external light during LED illumination which is illustrated in Fig. 6. For LED illumination, the 10 ml of cell suspension in TSB was transferred into a sterile petri dish (57 mm diameter) with 8 mm in depth and placed directly below the LED bulbs at a distance of 80–150 mm to adjust Photosynthetic Photon Flux Density (PPFD) of 300 μmol/m^2^s which is the optimum condition for LED illumination to lettuce in the plant factory^21,41^. The PPFD of each LED in the system was measured using a PPFD meter (PAR-100, J&C Technology, Gimcheon-si, Korea) at the same distance. The temperature of TSB in each LED system was monitored with a Fluke 5.4 thermocouple thermometer (Everett, WA, USA) during LED illumination for 4 h.

### Growth kinetics of *E. coli* O157:H7 under LED illumination

Ten milliliters of cell suspension in TSB was illuminated by 465, 520, and 625 nm LED for 24–30 h at the set temperature of 24.5 °C. Cell growth under LED illumination was monitored periodically by sampling at appropriate time intervals, diluting in 0.1% (wt/vol) peptone water (PW; Oxoid), and plating on tryptic soya agar (TSA; Oxoid). The cells grown under dark conditions (non-illuminated) at the set temperature of 25 °C served as a control in this study. The number of viable cells expressed as log CFU/ml was plotted against time. The growth curves were generated by fitting the data to the equation of Baranyi and Roberts (1994) using DMFit (https://browser.combase.cc/DMFit.aspx) and the growth parameters, namely, lag phase duration (LPD), specific growth rate (GR), doubling time (DT) and maximum population density (MPD), were calculated. Based on the growth curve, the time for the early stationary phase of cells under each LED illumination was also determined and the cells were collected for tolerance to each stress condition and RNA-seq analysis.

### Transcriptomic analysis of LED-illuminated *E. coli* O157:H7

Transcriptomic analysis was carried out in triplicate of LED-illuminated and non-illuminated control cells to better understand differences in the transcriptional response of *E. coli* O157:H7 among 465, 520, and 625 nm LEDs and non-illuminated control cells. Non-illuminated and illuminated *E. coli* O157:H7 cells for early stationary-phase were stabilized with an RNA protect Bacterial Reagent (Qiagen, Hilden, Germany) and total RNA was isolated using a RNeasy Mini kit (Qiagen) with a gDNA Eliminator spin column (Qiagen), according to the manufacturer’s instructions. The concentration and the purity of extracted RNA were determined using a NanoVue Plus Spectrophotometer (GE Healthcare, Little Chalfont, Buckinghamshire, UK) and the integrity was confirmed by agarose gel electrophoresis.

For library construction and sequencing, samples of total RNA were performed in triplicate in Macrogen Inc. (Seoul, Republic of Korea). Briefly, rRNA in total RNA was depleted with an Epicenter Ribo-Zero rRNA Removal Kit (Bacteria) (Illumina Inc., San Diego, CA, USA) and then sequencing libraries were constructed by a TruSeq Stranded Total RNA Sample Prep Kit (Illumina Inc.) according to the manufacturer’s instructions. Genomic data were generated on a HiSeq 4000 system (Illumina Inc.) using a paired-end protocol and a read length of 2 × 100 bp. The quality of the raw sequences was verified using FastQC software (version 0.11.7; Babraham Bioinformatics, Cambridge, UK). Before analysis, the raw paired-end reads were trimmed and quality filtered using a Trimmomatic program^42^ (version 0.38) and was generated by removing adapter sequences, low-quality bases (base quality length < 3 and sliding window size 4 and quality score 15), and short reads (< 36 bp). After quality control, the trimmed reads were mapped onto the *E. coli* O157:H7 reference genome (GenBank: GCF_000008865.2, NCBI: asm886v2) using a Bowtie program (version 1.1.2; http://bowtie-bio.sourceforge.net/index.shtml). The number of mapped reads to each gene was counted with an HTseq program (version 0.10.0; http://www-huber.embl.de/users/anders/HTSeq/doc/overview.html) to measure the expression. The differentially expressed genes (DEGs) assay was analyzed with reads per kilobase of transcript per million mapped reads (RPKM) method. Statistical analysis was performed with the fold change (FC), independent T-test, and hierarchical clustering. The conditions of |FC|≥ 2 and independent T-test raw *P*-value < 0.05 were selected. Hierarchical clustering analysis was carried out with Euclidean distances and complete lineage as measures of similarity of each gene in each sample. Principal component analysis (PCA) was performed by following the method described by Curiel et al.^43^.

### Transcriptomic functional analyses of LED-illuminated *E. coli* O157:H7 based on KEGG pathways

Functional genes of the LED-illuminated *E. coli* O157:H7 were identified from genomes using Prokka with default parameters^44^ and were functionally annotated using BlastKOALA (http://www.kegg.jp/blastkoala/)^45^. In each KEGG category, the transcriptional expression of the genes is shown as the sum of the read numbers per kilobase of each coding sequence per million mapped reads (RPKM) values of mRNA reads, assigned to each KEGG functional category. In addition, based on the KEGG Orthology (KO) numbers of the functional genes, metabolic pathways of the LED-illuminated cells and non-illuminated control cells of *E. coli* O157:H7 were generated in iPath v3 module (https://pathways.embl.de/)^46^. The transcriptional levels of the KEGG pathways of *E. coli* O157:H7 under LED illumination and non-illumination were represented relatively using different line thicknesses and color brightness based on the sum of the RPKM values of all the functional genes.

### Statistical analysis

All experiments were repeated in triplicate and data were presented as means ± standard deviation. SPSS version 25 (Statistical Package for Social Sciences, SPSS Inc, Chicago, USA) was used for statistical analysis. Significance was verified by one-way ANOVA followed by Duncan’s multiple range posthoc test. Significance was set at *P* < 0.05.

## Data Availability

The datasets generated and/or analysed during the current study are publicly available in the NCBI Sequence Read Archive (SRA) under Accession Numbers SRX18394425 to SRX18394436, (NCBI BioProject accession number PRJNA905884).

## References

[1] Liu Z (2021). Spectral design of light-emitting diodes for plant photosynthesis based on quantum dots. IEEE Access.

[2] Ray DK (2019). Climate change has likely already affected global food production. PLoS ONE.

[3] Gaupp F, Hall J, Hochrainer-Stigler S, Dadson S (2020). Changing risks of simultaneous global breadbasket failure. Nat. Clim. Chang..

[4] Song Y, Linderholm HW, Luo Y, Xu J, Zhou G (2020). Climatic causes of maize production loss under global warming in Northeast China. Sustainability.

[5] Nguyen TPL, Seddaiu G, Roggero PP (2019). Declarative or procedural knowledge? Knowledge for enhancing farmers’ mitigation and adaptation behaviour to climate change. J. Rural. Stud..

[6] Elahi E, Khalid Z, Tauni MZ, Zhang H, Lirong X (2021). Extreme weather events risk to crop-production and the adaptation of innovative management strategies to mitigate the risk: A retrospective survey of rural Punjab, Pakistan. Technovation.

[7] Würtele MA (2011). Application of GaN-based ultraviolet-C light emitting diodes: UV LEDs—for water disinfection. Water Res..

[8] Lee JY, Yang SY, Yoon KS (2021). Control measures of pathogenic microorganisms and shelf-life extension of fresh-cut vegetables. Foods.

[9] Ma G (2015). Effect of the combination of ethylene and red LED light irradiation on carotenoid accumulation and carotenogenic gene expression in the flavedo of citrus fruit. Postharvest. Biol. Technol..

[10] Wu MC (2007). A novel approach of LED light radiation improves the antioxidant activity of pea seedlings. Food Chem..

[11] Lee CL, Kim GH, Yoon KS (2021). Effects of combined aerosolization with ultraviolet C light-emitting diode on enterohemorrhagic *Escherichia coli* and *Staphylococcus aureus* attached to soft fresh produce. Foods.

[12] Sun H (2022). Regulation of flagellar motility and biosynthesis in enterohemorrhagic *Escherichia coli* O157:H7. Gut Microbes.

[13] Luna-Gierke RE (2014). Outbreaks of non-O157 Shiga toxin-producing *Escherichia coli* infection: USA. Epidemiol. Infect..

[14] Gobin M (2018). National outbreak of Shiga toxin-producing *Escherichia coli* O157:H7 linked to mixed salad leaves, United Kingdom, 2016. Eurosurveillance.

[15] Kozak GK, Donald DMAC, Landry L (2013). Foodborne Outbreaks in Canada Linked to Produce: 2001 through 2009. J. Food Protect..

[16] Lee HO (2009). Microbial contamination in a fresh-cut onion processing facility. Korean J. Food Preserv..

[17] Abadias M, Usall J, Anguera M, Solsona C, Viñas I (2008). Microbiological quality of fresh, minimally-processed fruit and vegetables, and sprouts from retail establishments. Int. J. Food Microbiol..

[18] Green A, Popović V, Warriner K, Koutchma T (2020). The efficacy of UVC LEDs and low-pressure mercury lamps for the reduction of *Escherichia coli* O157:H7 and *Listeria monocytogenes* on produce. Innovat. Food Sci. Emerg. Tech..

[19] Green A (2018). Inactivation of *Escherichia coli*, *Listeria* and *Salmonella* by single and multiple wavelength ultraviolet-light emitting diodes. Innovat. Food Sci. Emerg. Tech..

[20] Xiang Q (2020). Effect of UVC light-emitting diodes on apple juice: Inactivation of *Zygosaccharomyces rouxii* and determination of quality. Food Control.

[21] Ghate VS (2013). Antibacterial effect of light emitting diodes of visible wavelengths on selected foodborne pathogens at different illumination temperatures. Int. J. Food Microbiol..

[22] Kumar A (2016). Antibacterial efficacy of 405, 460 and 520 nm light emitting diodes on *Lactobacillus plantarum*, *Staphylococcus aureus* and *Vibrio parahaemolyticus*. J. Appl. Microbiol..

[23] Zhai Y (2021). Effects of UVC light-emitting diodes on inactivation of *Escherichia coli* O157:H7 and quality attributes of fresh-cut white pitaya. J. Food Meas. Charact..

[24] Maclean M, MacGregor SJ, Anderson JG, Woolsey G (2009). Inactivation of bacterial pathogens following exposure to light from a 405-nanometer light-emitting diode array. Appl. Environ. Microbiol..

[25] Luksiene Z (2003). Photodynamic therapy: Mechanism of action and ways to improve the efficiency of treatment. Medicina.

[26] Holland IB (2019). Rise and rise of the ABC transporter families. Res. Microbiol..

[27] Lewinson O, Livnat-Levanon N (2017). Mechanism of action of ABC importers: Conservation, divergence, and physiological adaptations. J. Mol. Biol..

[28] Moussatova A, Kandt C, O’Mara ML, Tieleman DP (2008). ATP-binding cassette transporters in *Escherichia coli*. Biochim. Biophys. Acta. Biomembr..

[29] Rees DC, Johnson E, Lewinson O (2009). ABC transporters: The power to change. Nat. Rev. Mol. Cell Biol..

[30] Li J, Liu D, Ding T (2021). Transcriptomic analysis reveal differential gene expressions of *Escherichia coli* O157:H7 under ultrasonic stress. Ultrason. Sonochem..

[31] Fujita Y, Matsuoka H, Hirooka K (2007). Regulation of fatty acid metabolism in bacteria. Mol. Microbiol..

[32] Farewell A, Diez AA, DiRusso CC, Nyström T (1996). Role of the *Escherichia coli fadR* regulator in stasis survival and growth phase-dependent expression of the *uspA*, *fad*, and *fab* genes. J. Bacteriol..

[33] Colin R, Ni B, Laganenka L, Sourjik V (2021). Multiple functions of flagellar motility and chemotaxis in bacterial physiology. FEMS. Microbiol. Rev..

[34] Colin R, Sourjik V (2017). Emergent properties of bacterial chemotaxis pathway. Curr. Opin. Microbiol..

[35] Milo R, Jorgensen P, Moran U, Weber G, Springer M (2009). BioNumbers–the database of key numbers in molecular and cell biology. Nucleic Acids Res..

[36] Ni B, Colin R, Link H, Endres RG, Sourjik V (2020). Growth-rate dependent resource investment in bacterial motile behavior quantitatively follows potential benefit of chemotaxis. Proc. Natl. Acad. Sci..

[37] Ni B (2017). Evolutionary remodeling of bacterial motility checkpoint control. Cell Rep..

[38] Federle MJ, Bassler BL (2003). Interspecies communication in bacteria. J. Clin. Invest..

[39] Sharma A, Singh P, Sarmah BK, Nandi SP (2020). Quorum sensing: Its role in microbial social networking. Res. Microbiol..

[40] Park H, Lee K, Yeo S, Shin H, Holzapfel WH (2017). Autoinducer-2 quorum sensing influences viability of *Escherichia coli* O157:H7 under osmotic and in vitro gastrointestinal stress conditions. Front. Microbiol..

[41] Ghate V, Zelinger E, Shoyhet H, Hayouka Z (2019). Inactivation of *Listeria monocytogenes* on paperboard, a food packaging material, using 410 nm light emitting diodes. Food Control.

[42] Bolger AM, Lohse M, Usadel B (2014). Trimmomatic: A flexible trimmer for Illumina sequence data. Bioinformatics.

[43] Curiel JA, Morales P, Gonzalez R, Tronchoni J (2017). Different non-Saccharomyces yeast species stimulate nutrient consumption in *S. cerevisiae* mixed cultures. Front. Microbiol..

[44] Seemann T (2014). Prokka: Rapid prokaryotic genome annotation. Bioinformatics.

[45] Kanehisa M, Sato Y, Morishima K (2016). BlastKOALA and GhostKOALA: KEGG tools for functional characterization of genome and metagenome sequences. J. Mol. Biol..

[46] Darzi Y, Letunic I, Bork P, Yamada T (2018). IPath3.0: Interactive pathways explorer v3. Nucleic Acids Res..

